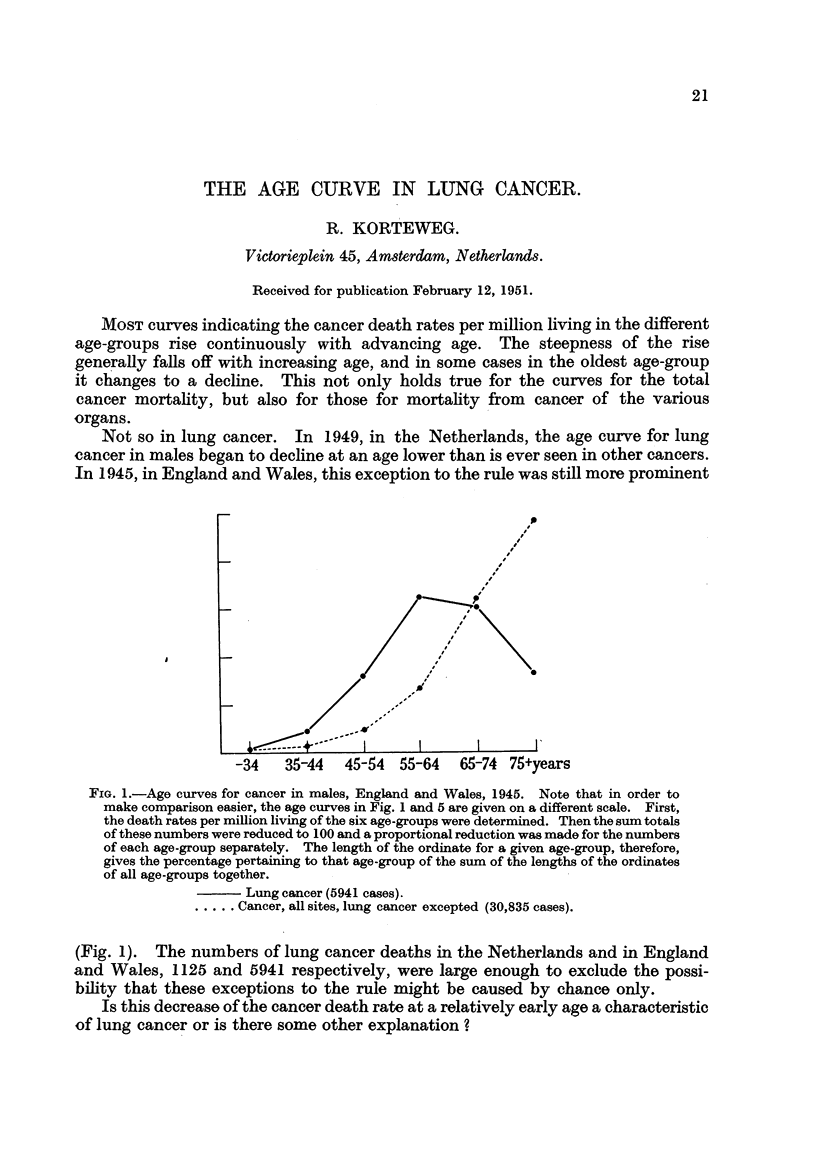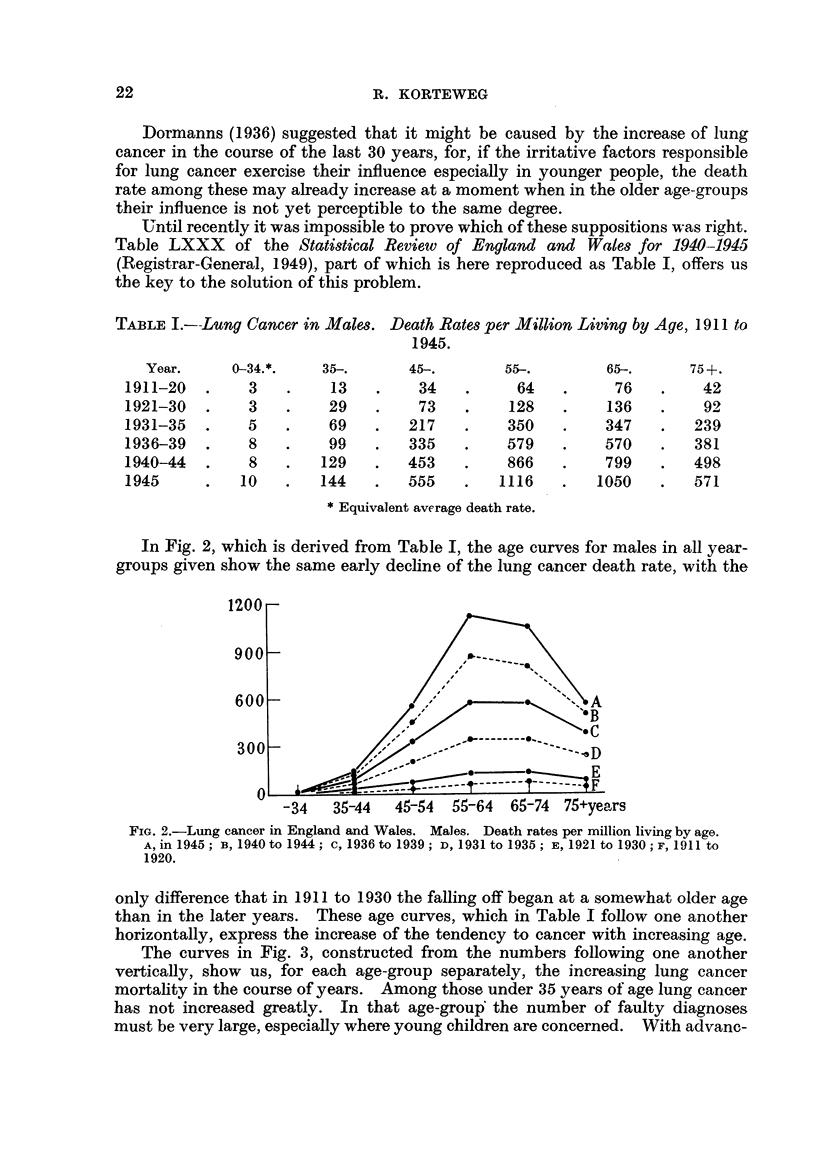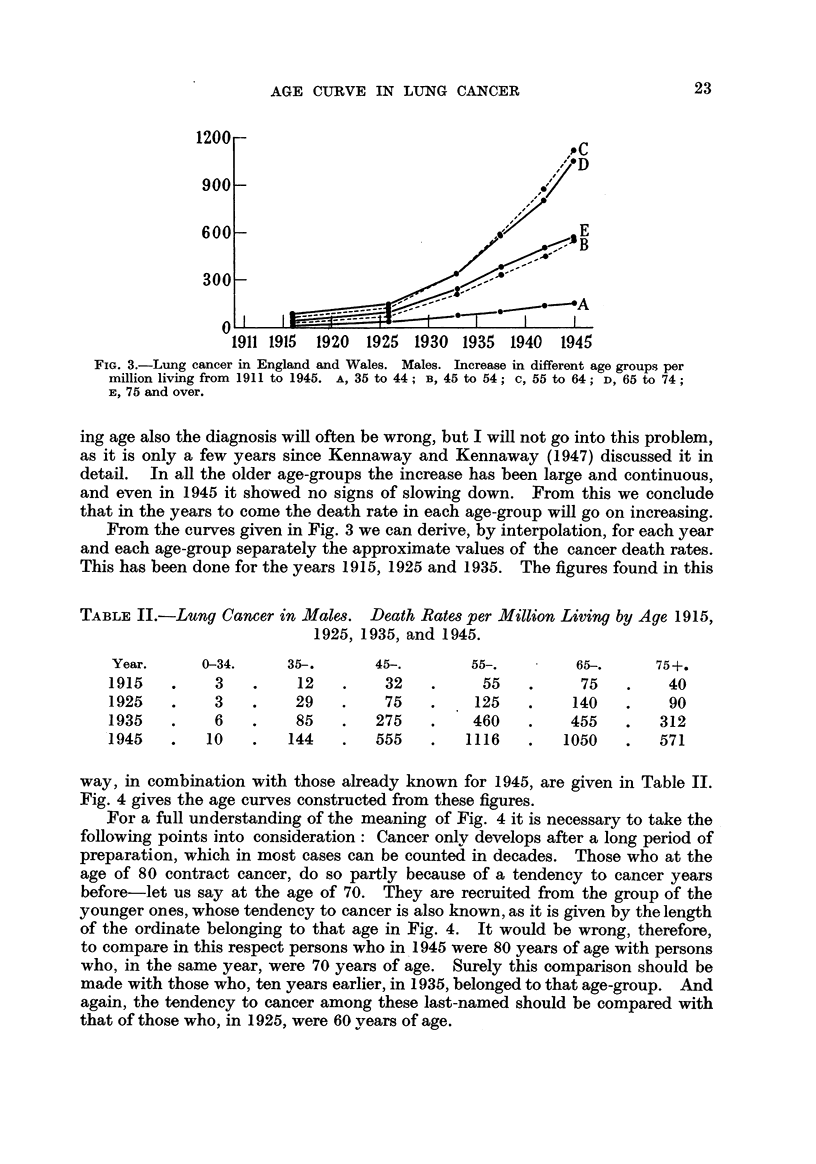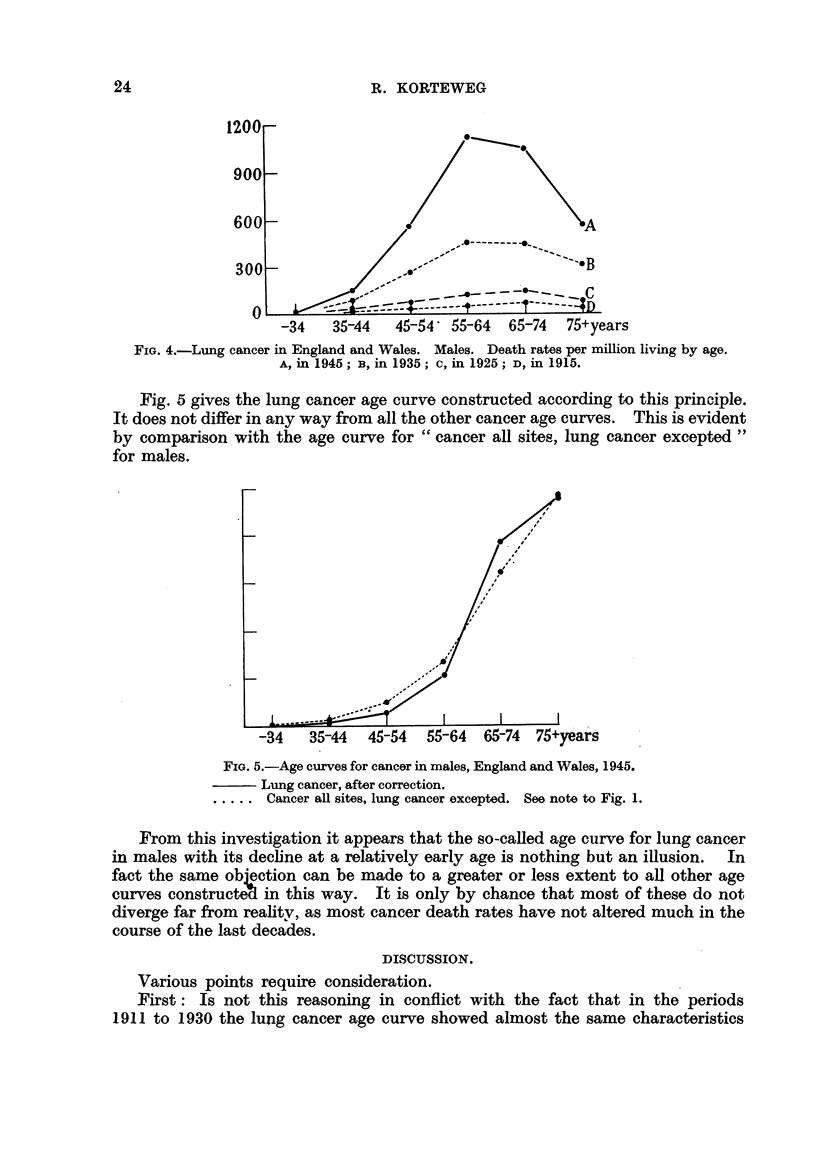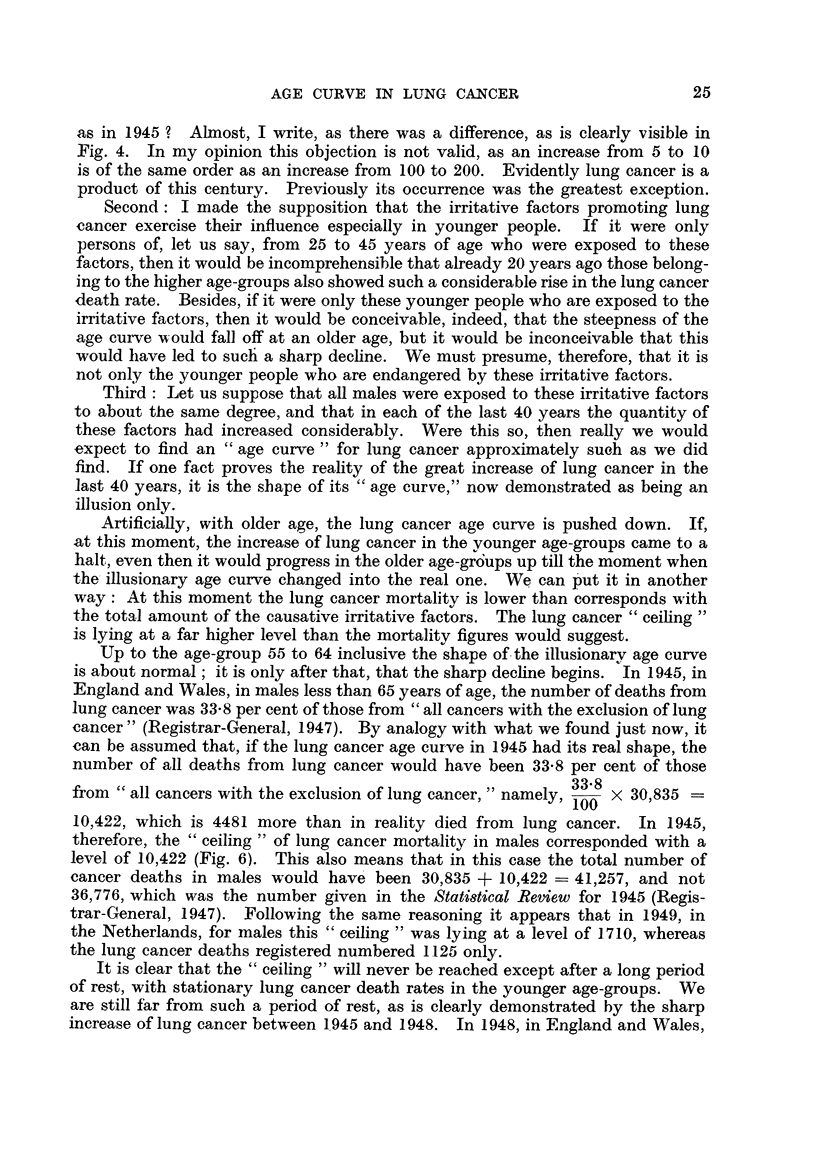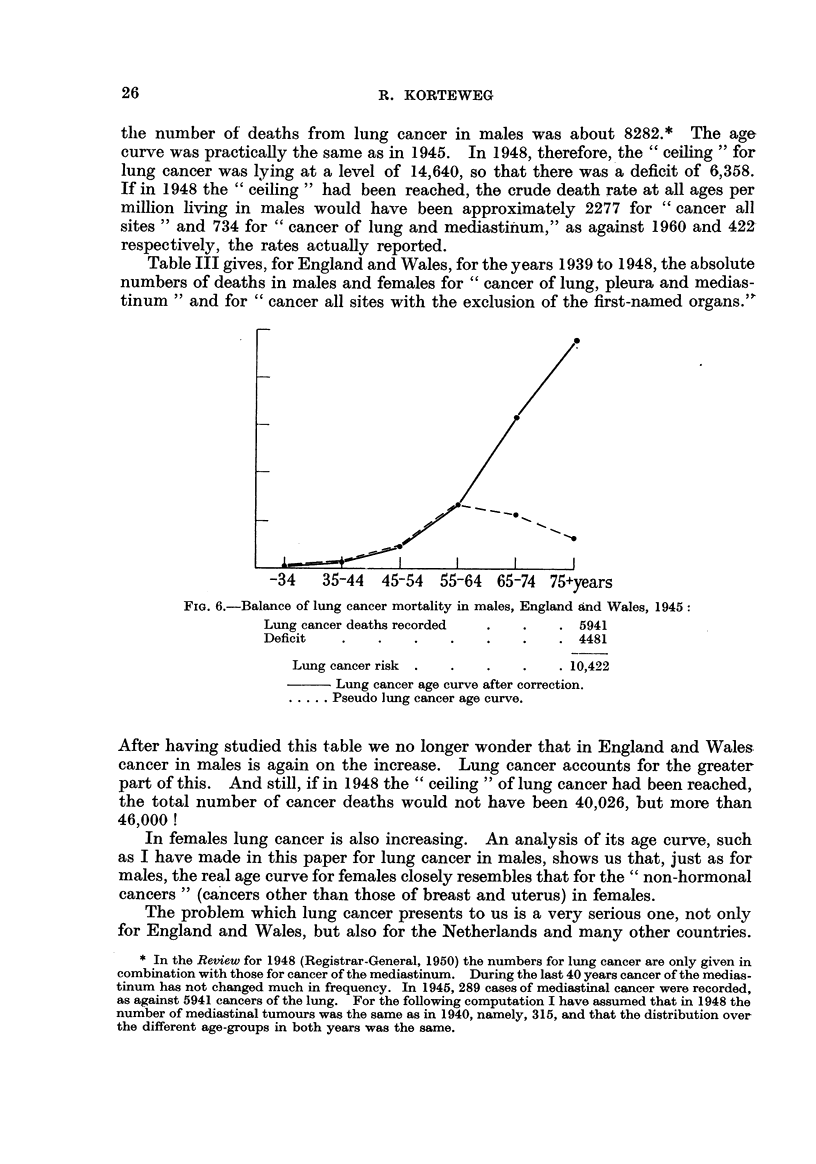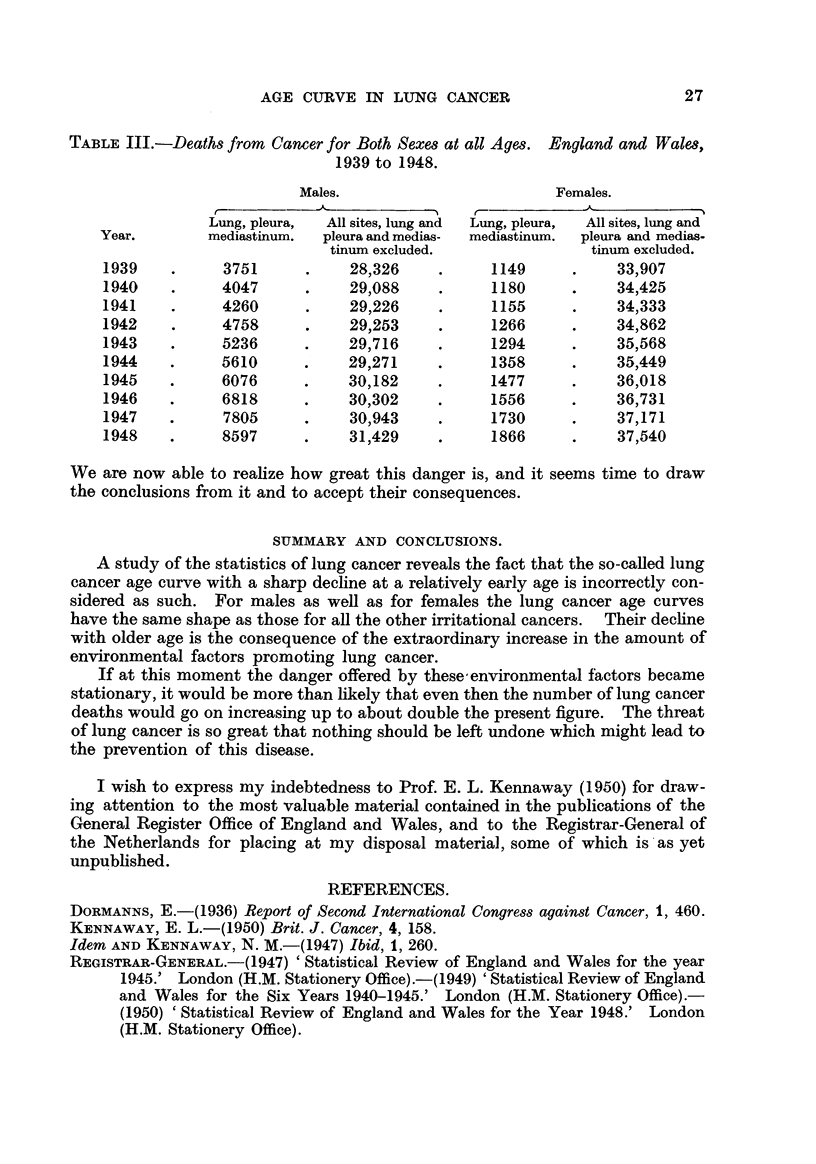# The Age Curve in Lung Cancer

**DOI:** 10.1038/bjc.1951.2

**Published:** 1951-03

**Authors:** R. Korteweg


### THE AGE CURVE IN LUNG CANCER.

R. KORTEWEG.

*Victorieplein 45, Amsterdam, Netherlands.*

Received for publication February 12, 1951.

MOST curves indicating the cancer death rates per million living in the different age-groups rise continuously with advancing age. The steepness of the rise generally falls off with increasing age, and in some cases in the oldest age-group it changes to a decline. This not only holds true for the curves for the total cancer mortality, but also for those for mortality from cancer of the various organs.

Not so in lung cancer. In 1949, in the Netherlands, the age curve for lung cancer in males began to decline at an age lower than is ever seen in other cancers. In 1945, in England and Wales, this exception to the rule was still more prominent

FIG. 1.—Age curves for cancer in males, England and Wales, 1945. Note that in order to make comparison easier, the age curves in Fig. 1 and 5 are given on a different scale. First, the death rates per million living of the six age-groups were determined. Then the sum totals of these numbers were reduced to 100 and a proportional reduction was made for the numbers of each age-group separately. The length of the ordinate for a given age-group, therefore, gives the percentage pertaining to that age-group of the sum of the lengths of the ordinates of all age-groups together.

——— Lung cancer (5941 cases).

. . . . . Cancer, all sites, lung cancer excepted (30,835 cases).

(Fig. 1). The numbers of lung cancer deaths in the Netherlands and in England and Wales, 1125 and 5941 respectively, were large enough to exclude the possibility that these exceptions to the rule might be caused by chance only.

Is this decrease of the cancer death rate at a relatively early age a characteristic of lung cancer or is there some other explanation ?

Dormanns (1936) suggested that it might be caused by the increase of lung cancer in the course of the last 30 years, for, if the irritative factors responsible for lung cancer exercise their influence especially in younger people, the death rate among these may already increase at a moment when in the older age-groups their influence is not yet perceptible to the same degree.

Until recently it was impossible to prove which of these suppositions was right. Table LXXX of the *Statistical Review of England and Wales for 1940–1945* (Registrar-General, 1949), part of which is here reproduced as Table I, offers us the key to the solution of this problem.

TABLE I.—*Lung Cancer in Males. Death Rates per Million Living by Age*, 1911 *to* 1945.

| Year. | 0–34.*. | 35–. | 45–. | 55–. | 65–. | 75+. |
|---|---|---|---|---|---|---|
| 1911–20 | 3 | 13 | 34 | 64 | 76 | 42 |
| 1921–30 | 3 | 29 | 73 | 128 | 136 | 92 |
| 1931–35 | 5 | 69 | 217 | 350 | 347 | 239 |
| 1936–39 | 8 | 99 | 335 | 579 | 570 | 381 |
| 1940–44 | 8 | 129 | 453 | 866 | 799 | 498 |
| 1945 | 10 | 144 | 555 | 1116 | 1050 | 571 |

* Equivalent average death rate.

In Fig. 2, which is derived from Table I, the age curves for males in all year-groups given show the same early decline of the lung cancer death rate, with the

FIG. 2.—Lung cancer in England and Wales. Males. Death rates per million living by age. A, in 1945 ; B, 1940 to 1944 ; C, 1936 to 1939 ; D, 1931 to 1935 ; E, 1921 to 1930 ; F, 1911 to 1920.

only difference that in 1911 to 1930 the falling off began at a somewhat older age than in the later years. These age curves, which in Table I follow one another horizontally, express the increase of the tendency to cancer with increasing age.

The curves in Fig. 3, constructed from the numbers following one another vertically, show us, for each age-group separately, the increasing lung cancer mortality in the course of years. Among those under 35 years of age lung cancer has not increased greatly. In that age-group the number of faulty diagnoses must be very large, especially where young children are concerned. With advanc-

Fig. 3.—Lung cancer in England and Wales. Males. Increase in different age groups per million living from 1911 to 1945. A, 35 to 44; B, 45 to 54; C, 55 to 64; D, 65 to 74; E, 75 and over.

ing age also the diagnosis will often be wrong, but I will not go into this problem, as it is only a few years since Kennaway and Kennaway (1947) discussed it in detail. In all the older age-groups the increase has been large and continuous, and even in 1945 it showed no signs of slowing down. From this we conclude that in the years to come the death rate in each age-group will go on increasing.

From the curves given in Fig. 3 we can derive, by interpolation, for each year and each age-group separately the approximate values of the cancer death rates. This has been done for the years 1915, 1925 and 1935. The figures found in this

Table II.—*Lung Cancer in Males. Death Rates per Million Living by Age* 1915, 1925, 1935, and 1945.

| Year. | 0–34. | 35–. | 45–. | 55–. | 65–. | 75+. |
|---|---|---|---|---|---|---|
| 1915 | 3 | 12 | 32 | 55 | 75 | 40 |
| 1925 | 3 | 29 | 75 | 125 | 140 | 90 |
| 1935 | 6 | 85 | 275 | 460 | 455 | 312 |
| 1945 | 10 | 144 | 555 | 1116 | 1050 | 571 |

way, in combination with those already known for 1945, are given in Table II. Fig. 4 gives the age curves constructed from these figures.

For a full understanding of the meaning of Fig. 4 it is necessary to take the following points into consideration: Cancer only develops after a long period of preparation, which in most cases can be counted in decades. Those who at the age of 80 contract cancer, do so partly because of a tendency to cancer years before—let us say at the age of 70. They are recruited from the group of the younger ones, whose tendency to cancer is also known, as it is given by the length of the ordinate belonging to that age in Fig. 4. It would be wrong, therefore, to compare in this respect persons who in 1945 were 80 years of age with persons who, in the same year, were 70 years of age. Surely this comparison should be made with those who, ten years earlier, in 1935, belonged to that age-group. And again, the tendency to cancer among these last-named should be compared with that of those who, in 1925, were 60 years of age.

FIG. 4.—Lung cancer in England and Wales. Males. Death rates per million living by age. A, in 1945 ; B, in 1935 ; C, in 1925 ; D, in 1915.

Fig. 5 gives the lung cancer age curve constructed according to this principle. It does not differ in any way from all the other cancer age curves. This is evident by comparison with the age curve for " cancer all sites, lung cancer excepted " for males.

FIG. 5.—Age curves for cancer in males, England and Wales, 1945.
——— Lung cancer, after correction.
. . . . . Cancer all sites, lung cancer excepted. See note to Fig. 1.

From this investigation it appears that the so-called age curve for lung cancer in males with its decline at a relatively early age is nothing but an illusion. In fact the same objection can be made to a greater or less extent to all other age curves constructed in this way. It is only by chance that most of these do not diverge far from reality, as most cancer death rates have not altered much in the course of the last decades.

### DISCUSSION.

Various points require consideration.

First : Is not this reasoning in conflict with the fact that in the periods 1911 to 1930 the lung cancer age curve showed almost the same characteristics as in 1945 ? Almost, I write, as there was a difference, as is clearly visible in Fig. 4. In my opinion this objection is not valid, as an increase from 5 to 10 is of the same order as an increase from 100 to 200. Evidently lung cancer is a product of this century. Previously its occurrence was the greatest exception.

Second : I made the supposition that the irritative factors promoting lung cancer exercise their influence especially in younger people. If it were only persons of, let us say, from 25 to 45 years of age who were exposed to these factors, then it would be incomprehensible that already 20 years ago those belonging to the higher age-groups also showed such a considerable rise in the lung cancer death rate. Besides, if it were only these younger people who are exposed to the irritative factors, then it would be conceivable, indeed, that the steepness of the age curve would fall off at an older age, but it would be inconceivable that this would have led to such a sharp decline. We must presume, therefore, that it is not only the younger people who are endangered by these irritative factors.

Third : Let us suppose that all males were exposed to these irritative factors to about the same degree, and that in each of the last 40 years the quantity of these factors had increased considerably. Were this so, then really we would expect to find an " age curve " for lung cancer approximately such as we did find. If one fact proves the reality of the great increase of lung cancer in the last 40 years, it is the shape of its " age curve," now demonstrated as being an illusion only.

Artificially, with older age, the lung cancer age curve is pushed down. If, at this moment, the increase of lung cancer in the younger age-groups came to a halt, even then it would progress in the older age-groups up till the moment when the illusionary age curve changed into the real one. We can put it in another way : At this moment the lung cancer mortality is lower than corresponds with the total amount of the causative irritative factors. The lung cancer " ceiling " is lying at a far higher level than the mortality figures would suggest.

Up to the age-group 55 to 64 inclusive the shape of the illusionary age curve is about normal ; it is only after that, that the sharp decline begins. In 1945, in England and Wales, in males less than 65 years of age, the number of deaths from lung cancer was 33·8 per cent of those from "all cancers with the exclusion of lung cancer" (Registrar-General, 1947). By analogy with what we found just now, it can be assumed that, if the lung cancer age curve in 1945 had its real shape, the number of all deaths from lung cancer would have been 33·8 per cent of those from " all cancers with the exclusion of lung cancer, " namely, $\frac{33{\cdot}8}{100} \times 30{,}835 =$ 10,422, which is 4481 more than in reality died from lung cancer. In 1945, therefore, the " ceiling " of lung cancer mortality in males corresponded with a level of 10,422 (Fig. 6). This also means that in this case the total number of cancer deaths in males would have been $30{,}835 + 10{,}422 = 41{,}257$, and not 36,776, which was the number given in the *Statistical Review* for 1945 (Registrar-General, 1947). Following the same reasoning it appears that in 1949, in the Netherlands, for males this " ceiling " was lying at a level of 1710, whereas the lung cancer deaths registered numbered 1125 only.

It is clear that the " ceiling " will never be reached except after a long period of rest, with stationary lung cancer death rates in the younger age-groups. We are still far from such a period of rest, as is clearly demonstrated by the sharp increase of lung cancer between 1945 and 1948. In 1948, in England and Wales, the number of deaths from lung cancer in males was about 8282.* The age curve was practically the same as in 1945. In 1948, therefore, the " ceiling " for lung cancer was lying at a level of 14,640, so that there was a deficit of 6,358. If in 1948 the " ceiling " had been reached, the crude death rate at all ages per million living in males would have been approximately 2277 for " cancer all sites " and 734 for " cancer of lung and mediastinum," as against 1960 and 422 respectively, the rates actually reported.

Table III gives, for England and Wales, for the years 1939 to 1948, the absolute numbers of deaths in males and females for " cancer of lung, pleura and mediastinum " and for " cancer all sites with the exclusion of the first-named organs."

FIG. 6.—Balance of lung cancer mortality in males, England and Wales, 1945 :

| | |
|---|---|
| Lung cancer deaths recorded . . . | 5941 |
| Deficit . . . . . . . . | 4481 |
| Lung cancer risk . . . . . | 10,422 |

——— Lung cancer age curve after correction.
. . . . . Pseudo lung cancer age curve.

After having studied this table we no longer wonder that in England and Wales cancer in males is again on the increase. Lung cancer accounts for the greater part of this. And still, if in 1948 the " ceiling " of lung cancer had been reached, the total number of cancer deaths would not have been 40,026, but more than 46,000 !

In females lung cancer is also increasing. An analysis of its age curve, such as I have made in this paper for lung cancer in males, shows us that, just as for males, the real age curve for females closely resembles that for the " non-hormonal cancers " (cancers other than those of breast and uterus) in females.

The problem which lung cancer presents to us is a very serious one, not only for England and Wales, but also for the Netherlands and many other countries.

* In the *Review* for 1948 (Registrar-General, 1950) the numbers for lung cancer are only given in combination with those for cancer of the mediastinum. During the last 40 years cancer of the mediastinum has not changed much in frequency. In 1945, 289 cases of mediastinal cancer were recorded, as against 5941 cancers of the lung. For the following computation I have assumed that in 1948 the number of mediastinal tumours was the same as in 1940, namely, 315, and that the distribution over the different age-groups in both years was the same.

TABLE III.—*Deaths from Cancer for Both Sexes at all Ages. England and Wales, 1939 to 1948.*

| Year. | Males. Lung, pleura, mediastinum. | Males. All sites, lung and pleura and mediastinum excluded. | Females. Lung, pleura, mediastinum. | Females. All sites, lung and pleura and mediastinum excluded. |
|---|---|---|---|---|
| 1939 | 3751 | 28,326 | 1149 | 33,907 |
| 1940 | 4047 | 29,088 | 1180 | 34,425 |
| 1941 | 4260 | 29,226 | 1155 | 34,333 |
| 1942 | 4758 | 29,253 | 1266 | 34,862 |
| 1943 | 5236 | 29,716 | 1294 | 35,568 |
| 1944 | 5610 | 29,271 | 1358 | 35,449 |
| 1945 | 6076 | 30,182 | 1477 | 36,018 |
| 1946 | 6818 | 30,302 | 1556 | 36,731 |
| 1947 | 7805 | 30,943 | 1730 | 37,171 |
| 1948 | 8597 | 31,429 | 1866 | 37,540 |

We are now able to realize how great this danger is, and it seems time to draw the conclusions from it and to accept their consequences.

### SUMMARY AND CONCLUSIONS.

A study of the statistics of lung cancer reveals the fact that the so-called lung cancer age curve with a sharp decline at a relatively early age is incorrectly considered as such. For males as well as for females the lung cancer age curves have the same shape as those for all the other irritational cancers. Their decline with older age is the consequence of the extraordinary increase in the amount of environmental factors promoting lung cancer.

If at this moment the danger offered by these environmental factors became stationary, it would be more than likely that even then the number of lung cancer deaths would go on increasing up to about double the present figure. The threat of lung cancer is so great that nothing should be left undone which might lead to the prevention of this disease.

I wish to express my indebtedness to Prof. E. L. Kennaway (1950) for drawing attention to the most valuable material contained in the publications of the General Register Office of England and Wales, and to the Registrar-General of the Netherlands for placing at my disposal material, some of which is as yet unpublished.

### REFERENCES.

DORMANNS, E.—(1936) *Report of Second International Congress against Cancer*, **1**, 460.

KENNAWAY, E. L.—(1950) *Brit. J. Cancer*, **4**, 158.

*Idem* AND KENNAWAY, N. M.—(1947) *Ibid*, **1**, 260.

REGISTRAR-GENERAL.—(1947) 'Statistical Review of England and Wales for the year 1945.' London (H.M. Stationery Office).—(1949) 'Statistical Review of England and Wales for the Six Years 1940–1945.' London (H.M. Stationery Office).—(1950) 'Statistical Review of England and Wales for the Year 1948.' London (H.M. Stationery Office).